# *Gloeothece* sp. as a Nutraceutical Source—An Improved Method of Extraction of Carotenoids and Fatty Acids

**DOI:** 10.3390/md16090327

**Published:** 2018-09-11

**Authors:** Helena M. Amaro, A. Catarina Guedes, Marco A. C. Preto, I. Sousa-Pinto, F. Xavier Malcata

**Affiliations:** 1 Interdisciplinary Centre of Marine and Environmental Research (CIIMAR), University of Porto, Terminal de Cruzeiros do Porto de Leixões, Avenida General Norton de Matos, S/N, P-4450-208 Matosinhos, Portugal; lena.amaro@gmail.com (H.M.A.); mcpreto@gmail.com (M.A.C.P.); ispinto@ciimar.up.pt (I.S.-P.); 2 Institute of Biomedical Sciences Abel Salazar (ICBAS), University of Porto, Rua Jorge Viterbo Ferreira no. 228, P-4050-313 Porto, Portugal; 3 FCUP—Faculty of Sciences, University of Porto, Rua do Campo Alegre s/n, 4169-007 Porto, Portugal; 4 LEPABE—Laboratory of Process Engineering, Environment, Biotechnology and Energy, Rua Dr. Roberto Frias s/n, P-4200-465 Porto, Portugal; fmalcata@fe.up.pt; 5 Department of Chemical Engineering, University of Porto, Rua Dr. Roberto Frias, s/n 4200-465 Porto, Portugal

**Keywords:** CPSE, pressurized liquid extraction, PUFA, antioxidant, cyanobacteria, microalga

## Abstract

The nutraceutical potential of microalgae boomed with the exploitation of new species and sustainable extraction systems of bioactive compounds. Thus, a laboratory-made continuous pressurized solvent extraction system (CPSE) was built to optimize the extraction of antioxidant compounds, such as carotenoids and PUFA, from a scarcely studied prokaryotic microalga, *Gloeothece* sp. Following “green chemical principles” and using a GRAS solvent (ethanol), biomass amount, solvent flow-rate/pressure, temperature and solvent volume—including solvent recirculation—were sequentially optimized, with the carotenoids and PUFA content and antioxidant capacity being the objective functions. *Gloeothece* sp. bioactive compounds were best extracted at 60 °C and 180 bar. Recirculation of solvent in several cycles (C) led to an 11-fold extraction increase of β-carotene (3C) and 7.4-fold extraction of C18:2 n6 t (5C) when compared to operation in open systems. To fully validate results CPSE, this system was compared to a conventional extraction method, ultrasound assisted extraction (UAE). CPSE proved superior in extraction yield, increasing total carotenoids extraction up 3-fold and total PUFA extraction by ca. 1.5-fold, with particular extraction increase of 18:3 n3 by 9.6-fold. Thus, CPSE proved to be an efficient and greener extraction method to obtain bioactive extract from *Gloeothece* sp. for nutraceutical purposes—with low levels of resources spent, while lowering costs of production and environmental impacts.

## 1. Introduction

A nutraceutical, has been defined as a food or a food product obtained from a natural source that, beyond nutrition, can also provide medicinal or health benefits in prevention or/and treatment of disease [1]. Both prokaryotic (cyanobacteria) and eukaryotic microalgae have gained a lot of attention in this regard in the last decade. They have been used in the human diet as nutritional supplements for hundreds of years but only in 1974 was the microalga *Spirulina* declared a potential food for the future by the United Nations World Food Conference [2]. Nowadays, *Chlorella* and *Spirulina* spp. are the species allowed for human consumption and widely traded, in Europe, being considered one of the most nutritious foods known to man [3]. Nonetheless, there are still hundreds of microalgal species that may serve as a potential source of valuable bioactive compounds, such as carotenoids and polyunsaturated fatty acids (PUFAs) [4]. Such compounds can be extracted from non-conventional microalgal species and applied directly in the nutraceutical industry, namely, in extracts form, thus circumventing the high costs associated with downstream purification processes required for pharmaceutical applications [5,6]. Additionally, interest in microalgal carotenoids and fatty acids has recently boomed, due to their potential therapeutic application in disease prevention—arising chiefly from recognized antioxidant properties [6,7,8,9]. As reviewed previously, said compounds have been described to possess antitumor, anti-inflammatory and antimicrobial features [10].

In microalgae, carotenoids and PUFA are generally located in the intracellular space, or accumulated in organelles (e.g., thylakoids), vesicles or in the cytoplasm itself. Hence, the presence of a cell wall surrounding the cells and a cytoplasmic membrane—both acting as semipermeable barriers—hinders extraction processes and thus increases the cost of extract production [11,12]. In fact, extraction of bioactive metabolites from microalgae may reach 40–60% of the final costs of production, depending on the biochemical characteristics and degree of purity intended [13]—and this may constrain economic exploitation of microalgae as feedstock for bioactive compound production. Thereof, optimization of extraction of microalgal compounds is urged, not only from an economic view but also from an ecological and environmental perspective.

Usually, the extraction of microalgal bioproducts is chiefly conducted from dried biomass with organic or aqueous solvents, depending on the polarity of the target compound [14,15]. Traditional solvent extraction methods are common in the chemical industry; however, they resort to large amounts of organic solvents, are labour-intensive and may expose extracts to excessive heat, light and oxygen—thus inducing isomerization and oxidation of labile compounds. Moreover, they require extra energy input to recover solvents and may even contaminate the algal extract, thus reducing and restricting options for their end uses [16]. In this regard, such new technologies as pressurized liquid extraction (PLE) have emerged and possess a number of advantages [17,18]. Moreover, typical PLE systems pressurize and accelerate passage of solvent through the matrix, hence improving speed and extraction efficiency of desired compounds. PLE also resorts to conventional solvents, at controlled temperature and pressure, entail less solvent and shorter extraction times and preserves samples in an oxygen- and light-free environment—so this method appears particularly suitable for the nutraceutical industry [19,20]. Moreover, application of pressure allows use of temperatures above solvent (atmospheric) boiling point, while reducing solvent surface tension and allowing a better penetration into matrix pores. This leads to matrix disruption that enhances compound mass transfer from sample to solvent, thus improving extraction efficiency [20]. Several studies have demonstrated the advantage of using high pressure and temperature with forced flow of solvent for extraction of natural compounds from solid and semi-solid matrices by PLE [21,22,23,24,25,26]. However, PLE still is expensive and requires dedicated infrastructure and operation that limit its application in natural product extraction. Additionally, most commercial equipment only allows static extraction; in other words, once equilibria between compounds bounded to the matrix and those that solubilized in solvent is reached, the extraction process efficiency drops dramatically [27]. Furthermore, the use of extreme temperatures in PLE—up to 135–200 °C—and pressures up to 200 bar or even higher [28], in photosynthetic organism materials, may promote the formation of pyropheophytins, a chlorophyll derivative not originally present in the source matrix but possessing antimutagenic features as reported for *Chlorella vulgaris* extracts [27].

Thereof, the focus of this study was the development of a prototype for continuous pressurized solvent extraction (CPSE), designed to improve microalgal bioactive compound extraction in an economical and environmental-friendly manner, essentially compatible with use by nutraceutical industry. Biomass from a scarcely studied prokaryotic microalga—*Gloeothece* sp.—was used as the model; recent advances have shown that its lipidic component exhibits interesting antioxidant capacity [29], as well as in previous studies [30,31]. The CPSE system was developed for moderate temperatures (30–70 °C) and pressures (70–260 bar), thus framing the range of operational temperatures and pressures targeted in large scale-up operation systems [16]. Moreover, this lab-scale prototype was built for flexibility, low cost and possible to scaled-up, in order to maximize extraction of compounds with known antioxidant capacity (namely carotenoids and fatty acids) from microalgal biomass.

## 2. Results and Discussion

The selection of extraction technique of bioactive metabolites is crucial for industrial purposes. Thus, establishing a profitable fast, efficient economic and green process is the ideal scenario. A way by which may be possible, is by following “Green Analytical Chemistry” (GAC) principles. This principal requires: (1) reduction of sample amount; (2) simultaneous extraction of several compounds; and (3) increase of automation [32]. An ideal pressurized liquid extraction for bioactive metabolites in the food industry, should use the minimum amount of food grade solvents and achieve a selective extraction of bioactive compounds—while preserving chemical structure. Furthermore, it should also display great versatility and efficiency, by modifying the physicochemical properties of solvents (e.g., density, diffusivity, viscosity and dielectric constant) through manipulation of pressure and/or temperature of solvent, thus leading to changes in solvating power [27]. Hence, the main purpose of our extraction system was to obtain a bioactive extract with the maximum content of carotenoids and/or fatty acids, with minimum use of such resources such as energy, solvents and feedstock.

### 2.1. Biomass Amount Optimization

The first step was to establish the minimum amount of biomass of *Gloeothece* sp. to be used. Three amounts of freeze-dried biomass were accordingly tested: 50, 100 and 150 mg. The ideal biomass amount should maintain the proportionality between biomass and targeted compounds in extract. Hence, such parameters such as extract mass yield, carotenoids content, PUFA content and antioxidant capacity were determined. For that, average values of flow and temperature were used—2 mL·min^−1^ and 40 °C, respectively. At this stage of optimization, (and until optimization of the ethanol volume was done at stage 4) and excess solvent volume was used. However, to establish that excess solvent volume, a kinetic assay was performed using average operational conditions of flow and temperature (2 mL·min^−1^ and 40 °C, respectively) and the maximum biomass tested—150 mg. Under a continuous solvent flow, several samples were collected in batches of 25 mL; and, after 2 consecutive batches deprived of material extracted, was found that a 150 mL was the solvent volume to be employed till in optimization of stages 1, 2 and 3 (data not shown).

Results of extract yield obtained at stage 1, expressed as mass ratio between extract/biomass, showed that there were not significant differences between the biomass amounts tested (*p* < 0.05). All amount of biomass attained ca. of 23 ± 0.7% of extraction, thus unfolding proportionality between biomass and mass of extract generated. In general, the same proportionality was observed in fatty acids and carotenoids content and antioxidant capacity—see Table 1. Note that for both carotenoids and PUFA quantification, only the most representative and bioactive ones were taken in consideration when comparing extraction performance, as listed in Table 1. To characterize extracts total antioxidant capacity, two total scavenging assays were performed: ABTS^+•^ and DPPH^•^. These two assays were chosen based on earlier experience [29], namely due to their different sensitivities to assay compounds—i.e., ABTS^+•^ for carotenoids and DPPH^•^ for PUFA. Upon inspection of Table 1, is clear that 50 mg of biomass is the most appropriate amount of biomass to be used, thus avoiding unnecessary wastage.

### 2.2. Solvent Flow-Rate Optimization

As stated before, the next stage was to optimize the solvent flow-rate—and 1 mL·min^−1^ (Q1), 2 mL·min^−1^ (Q2), 3 mL·min^−1^ (Q3) and 4 mL·min^−1^ (Q4) were tested. System pressure increased as flow increased and changed with temperature, so pressures of 72, 142, 210 and 260 bar were indeed reached, respectively, at 40 °C. Higher pressures facilitate transport of solvent to hard-to-reach corners, pores, surfaces and matrices [16,33]; this causes in matrix disruption, thus enhancing mass transfer of the target compound from the matrix to the solvent [20]. However, in terms of crude extract yield, there were no significant differences in the mass of extract obtained at the various solvent flows tested, 17.1 ± 0.95% (m_E_/m_B_) at Q1; 21.2 ± 2.1% (m_E_/m_B_) at Q2; 18.9 ± 1.3% (m_E_/m_B_) at Q3 and 19.5 ± 1.8% (m_E_/m_B_) at Q4.

**Figure 1:**
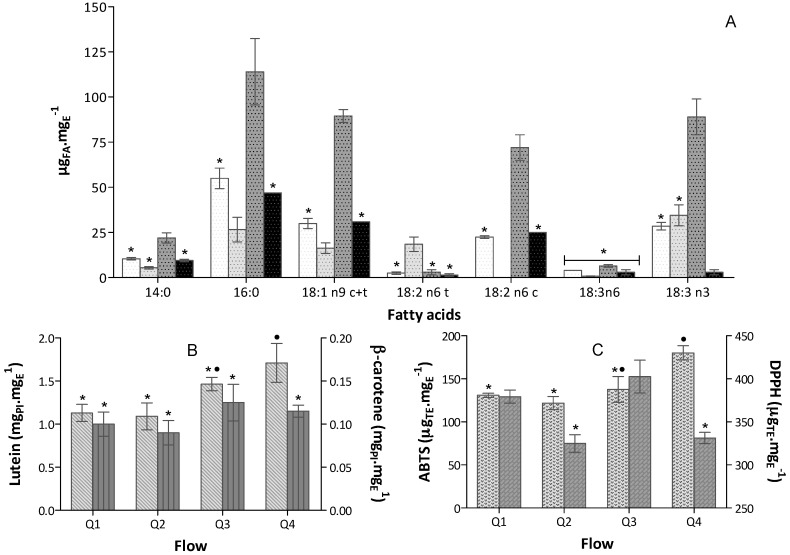
Biochemical profile of extracts (mean ± standard deviation) obtained from 50 mg-biomass in the various ethanol flows tested at 40 °C (bars for a common compound acid, without a common superscript, are significantly different, *p* < 0.05). (**A**) Fatty acid profile expressed as μg_FA_·mg_E_^−1^, (*n* = 6) obtained in 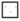 Q1 (1 mL·min^−1^); 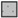 Q2 (2 mL·min^−1^); 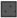 Q3 (3 mL·min^−1^) and 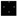 Q4 (4 mL·min^−1^); (**B**) Carotenoids content expressed in equivalent of PI (trans-β-Apo-8′-carotenal), mg_PI_·mg_E_^−1^ (*n* = 6) 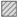 Lutein and 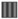 β-carotene; and (**C**) Antioxidant capacity expressed in trolox equivalent (TE) per extract mass, µ_TE_·mg_E_^−1^ (*n* = 9), obtained in 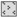 ABTS^+•^ and 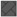 DPPH^•^ assays.

Several studies focusing on bioactive natural product extraction have pointed at the nil influence of extraction pressure; however, the only reason they set high pressure for extraction was to maintain its liquid state due the high solvent temperatures used (100–160 °C) with further influence not addressed [27,34]. Due to the existence of thermolabile compounds, only lower temperatures were tested in our study. Upon analysis of the biochemical profile of extracts obtained from 50 mg-biomass at the several solvent ethanol flows tested, at 40 °C (see Figure 1), it appears that flow-rate/pressure exerts a positive effect: this is particularly the case of Q3, in terms of fatty acid extraction (Figure 1A) and Q4 in terms of carotenoids (namely lutein)—when compared to Q1, about 3-fold and 1.3-fold increase, respectively, were observed (Figure 1B). Although selection of solvent is probably the most important stage in optimization of microalgal metabolite extraction by PLE [16], the use of higher pressure seems to increase extraction of lutein, as observed before [35]. With regard to β-carotene, our results showed that solvent flow rate (Q) did not cause any relevant effect (Figure 1B). A decreasing trend upon PUFA extraction was observed using Q4 (P of 260 bar) [35]. This can probably be explained by the increase of fluid density induced by pressure increases. A double effect was actually at stake: an increase in solvent solvating power and a reduced interaction between solvent and matrix—thus decreasing diffusion coefficient at higher density. This phenomenon has been previously described for other microalgae and metabolites [34,36].

As expected, the antioxidant capacity of the extracts obtained varied according to their content in carotenoids and fatty acids—see Figure 1. Hence, the antioxidant capacity as assessed by the ABTS^•+^ revealed no differences between extracts—except for Q4, which exhibited a higher concentration in antioxidant compounds, probably reflecting its higher lutein content. In terms of DPPH^•^ assay, Q3 and Q1 extracts attained the best results, probably for being richer in fatty acids.

According to effect upon compound extraction, Q3 flow rate was selected to proceed.

### 2.3. Temperature Optimization

Once biomass amount and solvent flow rate defined, the next stage was testing the influence of temperature on carotenoids and PUFA extraction.

Solvent pressure changes with increasing of temperature due a decrease of viscosity. Therefore, using 50 mg of biomass, at a solvent flow rate of Q3 (3 mL·min^−1^), pressures of 212, 210, 195, 180 and 168 bar were observed at solvent temperatures of 30, 40, 50, 60 and 70 °C, respectively. Temperature increases extraction potential of the solvent by accelerating diffusion rates [22]. Thermal energy also aids in overcoming the cohesive (solute-solute, i.e., lipid-lipid) interactions and adhesive (solute-matrix, i.e., lipids-cell matrix) interactions [16,33]. Increasing thermal energy also increases motion of, while molecular interactions associated with hydrogen bonds, *van der Waals* forces and dipole interactions concomitantly decreases—thus resulting in faster and easier extraction [33].

Our Results revealed that only at 70 °C was observed an increase of 8% in terms of mass extract yield, that is, 52% m_E_/m_B_ at 70 °C and average of 44.4 ± 2.8% m_E_/m_B_ at other temperatures (Figure S1).

**Figure 2:**
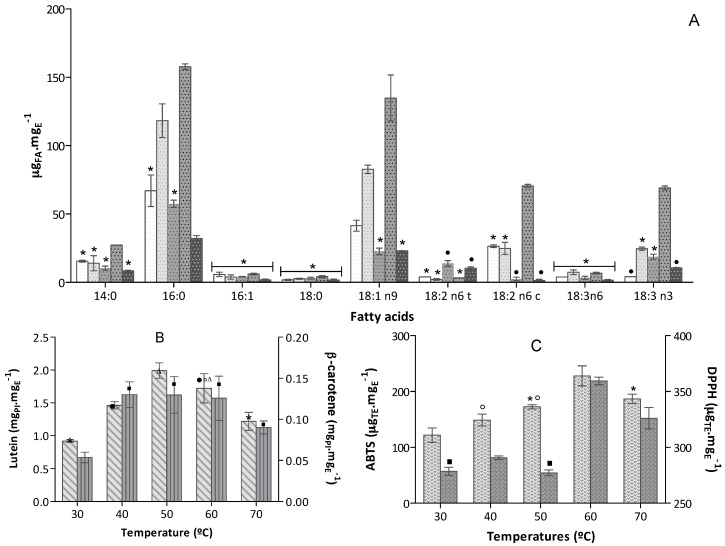
Extracts biochemical profile (mean ± standard deviation) obtained from 50 mg biomass, at ethanol flow of Q3, in the various extraction temperatures tested (bars without a common superscript are significantly different, *p* < 0.05). (**A**) Fatty acid profile expressed as μg_FA_·mg_E_^−1^ (*n* = 6) obtained at 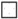 30 °C, 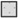 40 °C, 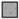 50 °C, 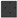 60 °C, 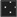 70 °C (bars for a common fatty acid, without a common superscript, are significantly different, *p* < 0.05); (**B**) Carotenoids content expressed in equivalent of PI mg_PI_·mg_E_^−1^ (*n* = 6) 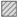 Lutein and 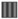 β-carotene; (**C**) Antioxidant capacity of the extracts expressed in trolox equivalent (TE) per extract mass, µ_TE_·mg_E_^−1^ (*n* = 9), obtained in 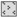 ABTS^+•^ and 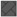 DPPH^•^ assays.

Analysing the biochemical profile of extracts obtained from 50 mg biomass, with an ethanol flow of Q3, at the several temperatures tested and in what concerns to fatty acid extraction (Figure 2A), extraction at 60 °C produced a better yield compared to the other temperatures tested. Comparing PUFA content results with those obtained at the lowest temperature tested, an increase of an average of 2.6-fold was observed in extraction of C16:0, 3.3-fold in C18:1 n9, 3.5-fold in C18:2 n6 trans, 2.7-fold in C18:3 n6 cis and 16-fold in C18:3 n3. As seen before by Iqbal et al. [16], diffusion rates increased roughly from about 2- to 10-fold when the temperature increased from 25 up to 100 °C. [16]. However, a linear extraction rate of lipids with temperature increase was not observed, indeed, at 70 °C, lipids extraction was significantly lower than at 60 °C. Although the use of higher temperatures has been claimed to enhance fatty acids extraction [16,25], the pressures used in said studies were lower than those in our work. The increase of temperature at 70 °C apparently reduced the solvent density considerably at 168 bar, thus decreasing the solvent-lipids contact—and resulting in net lower lipid mass transfer rates [37]. Lipids may also deteriorate by cleavage of carbon-oxygen bonds in fatty acids, due to sensitivity to temperature at the pressure used [16,38]. At 50 °C, an unexpected low extraction yield was attained. This may have been due to a complex interaction of non-equilibrium phenomena involving mass transfer due a change in solvent density because pressure, as observed before with microalgal carotenoid extraction [35].

Results of carotenoid recovery yield indicated that maximum extraction occurred within the range 50–60 °C for lutein, thus unfolding an increase in mass transfer rate with temperature—and indicating that 60 °C is the most appropriate temperature, as reported previously [35,36,39]. Nevertheless, temperature had no strong influence upon extraction of β-carotene; its concentration was similar (*p* < 0.05) in extracts obtained within of 40–70 °C but higher when compared the one obtained at 30 °C. Temperature affects viscosity and solubility of solvents but it may also promote isomerization and decomposition of labile target chemicals [22] which elucidate the slight decrease in lutein concentration observed at 70 °C (Figure 2B). Several pieces of evidence show that antioxidant capacity measured by ABTS^•+^ of such microalga extracts as *Haematococcus pluvialis*, seems to relate to free carotenoid content, mainly lutein [24,39,40]; whereas a decrease in antioxidant capacity seems relate to the lower carotenoid content of the extracts [39]. As expected, the antioxidant profile of ABTS^•+^ (Figure 2C) is similar to that obtained for carotenoids (Figure 2B), thus confirming that they are the compounds mainly responsible for such bioactivity.

On the other hand, fatty acids may contribute to the antioxidant activity, in addition to carotenoids [41]. This fact could be observed particularly at 60 °C, where the extract with higher content in carotenoids and in achieved also the better best antioxidant results in either DPPH^•^ or ABTS^•+^ assay (Figure 2C). However, other compounds with antioxidant capacity (not identified) are also co-extracted—like chlorophylls, phenolic compounds or other hydrophilic compounds and may also contribute to the antioxidant activity measured [42].

Therefore, the optimum temperature is ca. 60 °C, in agreement with previously studies [26].

### 2.4. Total Solvent Volume Optimization

The use of low solvent volumes in PLE extraction is one of its key points supporting applicability at industrial scale [25]—by reducing costs and being more environmental friendly. As stated before, the volume used so far was in excess; hence, so in a first attempt to reduce solvent volume and thus find the optimum ethanol volume, 150 mL of extract were collected in distinct and sequential fractions in order to determine compounds concentration and antioxidant capacity thereof. At the optimized conditions of temperature and solvent flow, 60 °C and Q3, the total extract volume (150 mL) was collected in 3 sequential fractions: 1st fraction of 12.5 mL (1F), 2nd fraction of 12.5 mL (2F) and 3rd fraction of 125 mL (3F). Results showed that total extract mass yields in 1F, 2F and 3F were 17.2 ± 0.1, 4.5 ± 0.4 and 6.9 ± 1.2% m_E_/m_B_, respectively. Hence, more than 60% of mass extract was concentrated in 1F—which corresponds to 8.3% of the initial volume of ethanol used so far, in terms of solvent volume.

**Figure 3:**
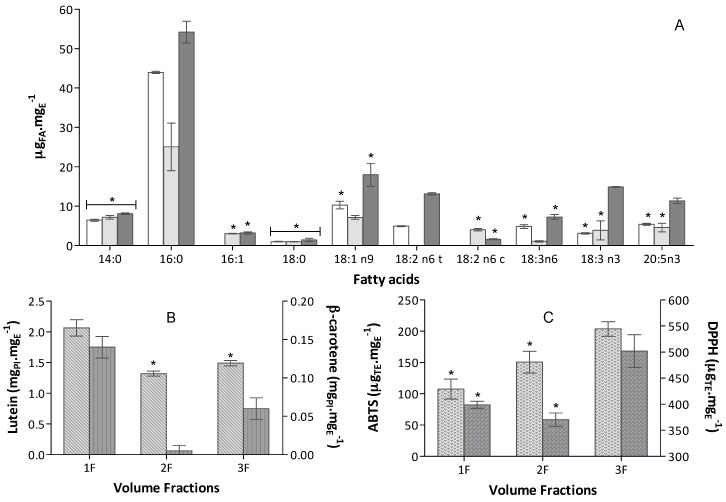
Extracts biochemical profile (mean ± standard deviation)obtained from 50 mg biomass, at 60 °C and ethanol flow of Q3, in the sequentially collected volume fractions (bars for the same assay, without a common superscript, are significantly different, *p* < 0.05); (**A**) Fatty acid profile expressed as μg_FA_·mg_E_ (*n* = 6) 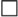 1F (12.5 mL), 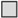 2F (12.5 mL), 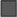 3F (125 mL); (**B**) Carotenoid content expressed in equivalent of PI mg_PI_·mg_E_^−1^ (*n* = 6) of 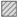 Lutein and 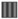 β-carotene; (**C**) Antioxidant capacity of the extract obtained in 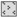 ABTS^+•^ and 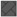 DPPH^•^ assays expressed in trolox equivalent (TE) per extract mass, µ_TE_·mg_E_^−1^ (*n* = 9).

Biochemical profile of extracts obtained from sequentially collected volume fractions, from 50 mg biomass, at 60 °C and solvent ethanol flow of Q3, are depicted in Figure 3. Analysing 1F fatty acids content of (Figure 3A), 29% of total fatty acids were herein obtained, as well as 42% of total extracted carotenoids, particularly β-carotene and lutein—which contained 70% of β-carotene and ca. 41% of lutein. The 2F composition (Figure 3A), although only 27% of carotenoids being extracted, 96% of it is lutein. In terms of fatty acids, 2F contain only ca. 23% of total fatty acids but a particular high content in 18:2 n6 c was extracted in this fraction—ca. 73.5% of total content on this PUFA. The main content of F3 is fatty acids (Figure 3A), ca. of 47% of the total fatty acids. Also, 3F is particularly rich in PUFA, with a content superior above 37%. In this fraction, the *trans* form of 18:2 n6 (ca. 72% of total content of this PUFA) was preferentially extracted. This observation is particularly interesting because it became possible to obtain different extracts with particular high content of the different isomers of the same fatty acid, namely18:2 n6 *cis* form in 2F and 18:2 n6 *trans* form in 3F. This may be of relevance due to the association of pro-inflammatory effects and promotion of coronary disease to intake of *trans* form of some PUFA [43]. In terms of carotenoids, F3 contains 30% of total carotenoids, being 94% which is lutein.

As expected, the composition of each fraction affected its antioxidant capacity. The different assays are sensible to different groups of compounds; the DPPH^•^ activity is better correlated to PUFA which content mostly contribute to this radical scavenging. Observing results, Figure 3, the profile in antioxidant capacity of each fraction follows it content in PUFA. 1F and 2F do not present statistical differences in antioxidant capacity for DPPH^•^ and contain very similar percentages of PUFA (around 35%) and 3F showed the highest antioxidant activity and the highest composition in PUFA, 50% of total fatty acids.

In terms of in ABTS^•+^ assay results, the fraction antioxidant activity was expected to follow carotenoid composition, 1F yielding higher results in terms of carotenoids. However, the best antioxidant activity in this assay was attained in 3F, being suspected that other compounds beyond carotenoids, such as some PUFA and phenolic compounds, may be contributing to total antioxidant capacity [44].

The study of composition of different extract fractions and bioactivity provided a comprehensive understanding of the potential application for nutraceutical purposes. This optimization stage showed that is possible to obtain extracts with different characteristics and to perform multiple compound extractions within the same procedure. The 1F is highly concentrated in lutein and β-carotene; 2F is particularly rich in lutein and 18:2 n6 c, so it is easier to purify this carotenoid (if that is the purpose); and 3F contains particular high antioxidant activity and high content in fatty acid.

#### Cycles of Solvent Recirculation

The extraction process depends on the time needed to reach equilibrium between the compound concentration in the sample matrix and the solvent [26]. Therefore, in an attempt to optimize the extraction of lipid component and reduce volume of solvent, the contact between solvent and matrix was increased by making the recirculation of the 1F for several cycles. Each time the solvent volume of 1F (12.5 mL) passes through the column corresponded to 1 cycle of recirculation (1C); and 1C takes 4 min. Hence, 1F recirculation was tested from 2 to 5 cycles (C). Results of % mass of extract obtained per mass of biomass (m_e_/m_b_) in the former section showed that 1F (=1C) was not saturated. It was observed an increase of 21% in terms of m_E_/m_B_ when recirculation was up to 2 or 3 cycles and an increase of 65% of m_E_/m_B_ when recirculation was up to 4 or 5 cycles (Figure S2 of Supplementary Data).

**Figure 4:**
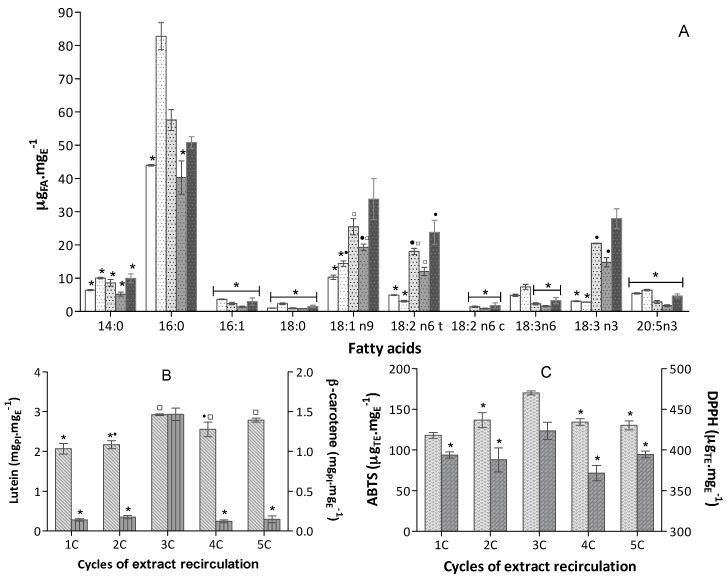
Extracts biochemical profile (mean ± standard deviation) obtained from 50 mg biomass, at 60 °C and ethanol flow of Q3, in in tested cycles of recirculation (bars for a common carotenoid, without a common superscript, are significantly different *p* < 0.05). (**A**) Fatty acid profile expressed as μg_FA_· mg_E_ (*n* = 6) obtained in 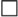 1C (4 min), 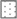
2C (8 min), 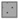
3C (12 min), 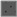
4C (16 min), 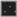
5C (20 min), (bars for a common fatty acid, without a common superscript, are significantly different, *p* < 0.05); (**B**) Carotenoid content expressed in equivalent of PI, mg_PI_·mg_E_^−1^ (*n* = 6) of 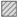 Lutein and 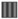 β-carotene); (**C**) Antioxidant capacity of the extracts obtained in 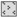 ABTS^+•^ and 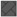 DPPH^•^ assays expressed in trolox equivalent (TE) per extract mass, µ_TE_·mg_E_^−1^ (*n* = 9).

As expected, the fatty acid concentration depicted in Figure 4A seems to increase with the number of cycles. Compared to 1C, fatty acids had the following average extraction improvement using 5C: 3-fold for 18:1 n9, 5-fold for C 18:2 n6 t and 9-fold for C18:3 n3 (Figure 4A). Although ASE systems usually work in sequential cycles of static volume, this effect was observed before [25] in fatty acid recovery.

The aforementioned phenomenon was also observed in carotenoid extraction: lutein content increased about 1.4-fold, from 1C to 3C. In the case of β-carotene, the increase was significantly more pronounced; it had an increase of 10-fold from 1C to 3C. The yield of carotenoids increased at 12 min (3C) of extraction; with additional cycles, the extraction is most likely desorption/diffusion controlled, as pointed out [20]. A too long extraction may also induce degradation of more sensitive carotenoids [20], as observed in β-carotene extraction in cycles longer than 3C (Figure 4B).

In terms of antioxidant capacity, using the in ABTS^•+^ assay, the increase of cycle of recirculation followed increase of carotenoid content of extracts, more precisely lutein content, thus proving that these carotenoids had a more pronounced contribution for such a bioactivity. In terms of DPPH^•^ assay, in general, the profile of antioxidant capacity follows the PUFA profile along the sequence of cycles of recirculation. However, it was expected that antioxidant activity of 5C be higher due the high concentration in PUFA. Carotenoids may also have some influence in this antioxidant assay and degradation of β-carotene may have also contributed to DPPH^•^ results in 5C.

### 2.5. Comparison of Lab-Made CPSE System with Ultrasound Assisted Extraction

Common PLE has been widely compared to other extraction techniques usually used in the industry to extract antioxidant compounds such as maceration, ultrasound assisted extraction (UAE) and Soxhlet extraction [45]. Advantages of PLE arise chiefly from lower volume, shorter extraction time and potential for automation [27]. In this work, a classic UAE was used for comparison and validation of results obtained with our CPSE system—carefully conducted to avoid isomerization and degradation of compounds. Towards this goal, the extraction conditions were the same those optimized in CPSE—both in terms of biomass amount (50 mg) and solvent volume (12.5 mL). Results presented in Table 2 compare results of UAE with the ones obtained with optimized CPSE. In overall, it was observed that compound concentration in CPSE system is generally superior to that obtained in UAE [42,46]. While carotenoids content is significantly greater when obtained in CPSE (about 2.3-fold for lutein and 15-fold for β-carotene), UAE attained the same or better yields of fatty acids extraction. The exception was observed only in the extraction of C16:0 and two PUFA (C18:1 n9 and C18:3 n3)—with extraction yields of an average of 1.3-fold, 1.4-fold and 9.6-fold higher, respectively. This might in fact be considered as an advantage of CPSE over UAE, once the system developed with 5C seems to be particular by selective to these two bioactive PUFA. Their selectivity of extraction, together with the superior results in carotenoids extraction yields, justifies the better results in terms of antioxidant capacity.

Therefore, CPSE extraction system offers the advantage of allowing better extraction yields, in a shorter time (about 3.8-fold less) and in a single step.

## 3. Materials and Methods

### 3.1. Microalga Source and Biomass Production

*Gloeothece* sp. (ATCC 27152), obtained from ATCC (American Type Culture Collection, Manassas, VA, USA), was produced in batch mode in 5 L-flasks containing 4.5 L of Blue Green medium (BG11) [47] buffered with Tri-(hydroxymethyl)-aminomethane hydrochloride (Tris-HCl) 25 mM, at 25 °C and pH 8. Continuous illumination was provided via fluorescent BIOLUX lamps (250 µmol_photon_·m^−2^·s^−1^) and air was bubbled at a flow rate of 0.5 L·min^−1^. To ensure the microalga was in the exponential growth phase, a pre-inoculum at an initial optical density of 0.1 at 680 nm was used for 10 days in 800 mL of the same medium. After 14 days of growth, biomass was centrifuged at 4000 rpm for 10 min and biomass freeze-dried and stored under nitrogen in a desiccator prior to utilization.

### 3.2. Continuous Pressurized Solvent Extraction

**Figure 5:**
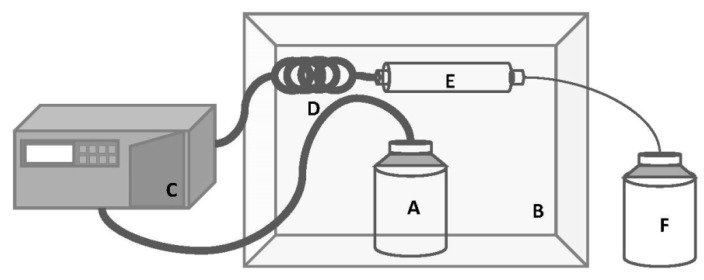
Scheme of the lab continuous pressurized solvent extraction (CPSE) system. This system is composed by a (**A**) solvent reservoir; (**B**) temperature controlled chamber; (**C**) HPLC solvent injection pump; (**D**) pre-heating coil; (**E**) extraction column; and (**F**) extract reservoir.

The lab-made continuous pressurized solvent extraction system (CPSE) was designed (and built) to allow a pressurized solvent pass through a column containing microalgal biomass, at a pre-set temperature. As depicted in Figure 5, it is mainly composed of an HPLC solvent injection pump (Hitachi L-2130, Tokyo, Japan) which pressurizes solvent at a set flow-rate (between 0.1–10 mL·min^−1^) and pressure (up to 360 bar); an extraction hollow column filled with microalgal biomass and an excipient (Ottawa sand); and a temperature-controlled chamber, which allows the system to be kept at the desired temperature.

The solvent reservoir was kept at the chosen temperature and pumped, at a set flow rate, to a stainless pre-heating coil, 1 m-long and 1 mm for internal diameter—which guarantees that solvent is at the chosen temperature before entering the extraction column. The extraction column placed inside the temperature-controlled chamber is 15 cm-long and has 50 mm of internal diameter, being closed with filter end fittings. The exit tube was 2 m-long, with an internal diameter of 50 µm (which keeps the system pressurized) and ends in the extract reservoir. In order to avoid leaks, the whole system was tested at the maximum operating pressures and temperatures. Furthermore, the system allows flushing of solvent in the tubes at the end of each assay, without the need for any inert gas (e.g., N_2_). As the entire system is closed, it avoids contact with O_2_. Between runs, the entire system was cleaned with fresh solvent to avoid any remaining extract becoming a carryover.

In each assay, the extraction column was filled in consecutive layers with inert Ottawa sand, *Gloeothece* sp. freeze-dried biomass and another layer of Ottawa sand. To guarantee a homogeneous particle size (in order to avoid diffusion paths), *Gloeothece* sp. freeze-dried biomass was standardized [34].

To assure that the whole system remains at constant temperature, solvent, pre-heating coil and column were placed in the temperature-controlled chamber for at least 5 min for pre-heating.

In order to summarize all optimization stages, tested conditions are depicted below in Table 3.

#### Extraction Conditions Optimization

The operation conditions of this CPSE system were optimized sequentially along four stages: (1) amount of biomass in extractor; (2) solvent flow/pressure (mL·min^−1^); (3) temperature (°C); and (4) total volume of solvent used (mL). As the optimization stages progressed, the conditions were redefined based on the results obtained at so far; the conditions were there generating an extract with higher lipidic component (carotenoids and PUFA) and better total antioxidant capacity.

Selection of the correct solvent is one of the most crucial factors affecting pressurized extraction. The targeted compounds were fatty acids and carotenoids; and based on an earlier study [29] and studies elsewhere [20,25,39,42], ethanol was selected. Besides its relatively low environmental impact, it has a positive net energy balance—and a generally recognized as safe (GRAS) status—an extra advantage for its applicability by the nutraceutical industry [20].

To establish the best biomass amount to perform extraction, 50, 100 and 150 mg of freeze-dried biomass were assayed (at medium-low temperature, 40 °C and ethanol flow rates of 2mL·min^−1^, respectively). The influence of solvent flow/pressure was studied using the previous selected biomass amount; hence, four different flow rates were tested—1, 2, 3 and 4 mL·min^−1^. Once the best combination of biomass amount and solvent flow was attained, several system temperatures were tested, viz. 30, 40, 50, 60 and 70 °C, reaching pressures of 212, 210, 195, 180 and 168 bar, respectively.

Until this point, all solvent used was circulating in an open continuous flow; to reduce use of solvent volume, this condition was optimized as last stage. Therefore, the extract was collected first in several fractions along the extraction process—and the content in PUFA, carotenoids and antioxidant capacity were determined in each fraction. Determination of minimum solvent volume corresponds to the cumulative volume of the fraction that contained the major amount of lipidic component and antioxidant capacity. In order to improve extraction efficiency, the minimum volume found was employed in recirculation mode in the CPSE system, connecting the extracts reservoir to the inlet solvent injection pump and making it pass again through the column. In this way, each time the solvent passed through the column defined one cycle—so, several cycles, of solvent recirculation were tested: 2, 3, 4 and 5 cycles which correspond to 4, 8, 12, 16 and 20 min. By the end of each cycle, the system was purged, the column detached and the system closed and submitted to a cleaning cycle with hot solvent for 5 min. All extractions were performed in triplicate, extracts were dried and maintained under N_2_ atmosphere till analysis. Aliquots of each extract were used to determine contents in fatty acids and carotenoids, as well as their antioxidant capacity.

### 3.3. Ultrasound Assisted Extraction

Ultrasound-assisted extraction was performed with 50 mg of freeze dried biomass and sequential addition of 2 mL of ethanol, until a final volume of 12.5 mL. In the first addition of solvent, cells were submitted to disruption by applying 15 min of continuous sonication at 45 KHz (USC100T VWR ultrasonic bath). Between each addition, the extract was stirred for 20 min at 250 rpm, centrifuged at 20,000 rpm for 5 min and the supernatant (extract) collected and stored at 4 °C. To completely remove cells debris, extracts were filtered through 0.45 µm pore size and then stored under nitrogen, at −20 °C in darkness, prior to analysis.

### 3.4. Antioxidant Scavenging Capacity Assessment of Extracts

#### 3.4.1. ABTS^+•^ Scavenging Capacity

An aliquot of each extract obtained through the CPSE system was evaporated and the residue re-suspended in ethanol:water, 50:50 (*v*/*v*) to a final concentration of 1 mg·mL^−1^. The ABTS^+•^ radical-scavenging capacity of the extracts was assessed in triplicate, as described elsewhere [23,30]. For quantification, a calibration curve using a known antioxidant—Trolox, was prepared, so antioxidant capacity of extract was expressed as equivalents of Trolox (µ_TE_·mg_E_^−1^).

#### 3.4.2. DPPH^•^ Scavenging Capacity

An aliquot of each extract was likewise evaporated and the residue resuspended in methanol to a final concentration of 5 mg·mL^−1^. In this spectrophotometric assay, the scavenging reaction was measured after incubation for 30 min of 1 mL DPPH^•^ with 125 μL of sample, as described elsewhere [29]. Measures were performed at 515 nm and quantification was performed as described above, being antioxidant capacity of extracts expressed in Trolox equivalents (µ_TE_·mg_E_^−1^).

### 3.5. Compound Identification

#### 3.5.1. Determination of Carotenoid Profile

To identify and then quantify carotenoids (including β-carotene and lutein, in particular), high-performance liquid chromatography (HPLC) was employed as analytical technique. An aliquot from each extract was evaporated and suspended to a concentration of 15 mg·mL^−1^ and β-apo-carotenol (Sigma) was used as an internal standard. The carotenoid profile was produced in a Merck-Hitachi HPLC system, equipped with a Diode Array Detector (DAD) Merck-Hitachi L-7450—to resolve, detect and identify the various chemical compounds of interest. The absorption spectra were recorded between 270 and 550 nm. The stationary-phase was a 4 × 250 mm Purospher Star RP-18e (5 μm) column (Merck). The mobile-phase was constituted by solvent A—ethyl acetate and solvent B—acetonitrile/water at 9:1 (*v*/*v*), both from VWR, at various volumetric ratios along elution time, for an overall flow rate of 1 mL min^−1^. The following gradient was used: 0–31 min (0–60% A); 31–46 min (60% A); 46–51 min (60–100% A); 51–55 min (100% A); 55–60 min (100–0% A); and 60–65 min (0% A). The elution times of the chromatographic standards were: lutein 14.4 min and β-carotene 34.4 min. Standards were purchased in CarotNature, Lutein (No. 0133, Xanthophyll, (3R,3′R,6′R)-β,ε-Carotene-3,3′-diol with 5% Zeaxanthin and purity of 96%), β-carotene (No. 0003, β, β -Carotene with 96% purity) and β-apo-carotenol (No. 0482, 8′-Apo- β -caroten-8′-al with 97%, purity). Identification was by comparison of retention times and UV–visible photo-diode array spectra, following a previous procedure [40].

#### 3.5.2. Determination of Fatty Acid Profile

Fatty acid methyl esters were produced from 5 mL evaporated aliquot of each extraction obtained by direct transesterification—according to the acidic method described by Lepage and Roy [48], after modifications introduced by Cohen et al. [49] using heptadecanoic (C17:0) acid as an internal standard and acetyl chloride as a catalyst. Esters were analysed in a GC Varian Chromapack CP-3800 gas chromatograph (GC), using a flame ionization detector and quantified with the software Varian Star Chromatography WorkStation (Version 5.50). A silica CP-WAX 52 CB (Agilent) column was used and helium was employed as carrier gas in splitless mode. Injector and detector were maintained at 260 and 280 °C, respectively, and the oven heating program started at 100 °C holding this temperature for 5 min. Temperatures increased till 180 °C, at a rate of 6 °C min^−1^ and again till 200 °C at 2 °C min^−1^. Temperature was continuously increased till 205 °C but at a slower rate of 0.5 °C min^−1^ and then faster at 1 °C min^−1^ till 230 °C. Finally, maximum temperature was reached 233 °C at a rate of 0.5 °C min^−1^. Chromatographic grade standards of fatty acids in methyl ester form CRM47885 (Supelco) were used for tentative identification, based on retention times: C13:0, C14:0, C14:1, C15:0, C15:1, C16:0, C16:1, C17:0, C17:1, C18:0, C18:1 n9-cis + trans, C18:2 n6, C18:2 n6 c, C18:3 n6, C18:3 n3, C20:0, C20:1, C20:5 n3, C21:0, C22:0, C22:2, C22:1 n9. The mean of the results from the aforementioned chemical assays was used as a datum point.

### 3.6. Statistical Analysis

The experimental data were analysed using GraphPad Prism v. 5.0. A first diagnostic unfolded a non-normal distribution of the data, so 1-way ANOVA with Tukey’s multi-comparison test was used to assess variances between different extract in terms of carotenoid content and antioxidant capacity; and two-way ANOVA with the same multi-comparison test in fatty acid content for extraction conditions. Since each datum point had been replicated, a representative measure of variability was available in all cases to support said statistical analyses.

## 4. Conclusions

Our low cost, laboratory scale CPSE system proved versatile and effective in obtaining a bioactive extract rich in carotenoids and PUFA for nutraceutical purposes. The optimum conditions found, in terms of temperature and pressures were 60 °C and 180 bar. These values are lower than those used by competing PLE methods, which means lower need of energy and thus lower cost.

It is possible to obtain bioactive extract with different compositions, by collecting sequential fractions of the extract—thus reinforcing the versatility of the system developed and making it particularly attractive for nutraceutical purposes.

Furthermore, the possibility of operating in a continuous closed mode and with a GRAS solvent, makes it possible to optimize the extraction yield of bioactive compounds in environmental friendly way and with the use of fewer resources (biomass and solvent) thus lowering costs and environmental impact. Therefore, with CPSE makes it possible to generate antioxidant extract rich in carotenoids (using 3 cycles of ethanol recirculation) or fatty acids (5 cycles of ethanol recirculation).

When compared to conventional methods, namely ultrasound assisted extraction (UAE), our CPSE proved more efficient in terms of bioactive compound extraction (carotenoids and PUFA).

Upon eventual scale-up, this system appears promising. This preliminary study was aimed at contributing to more efficient and proper exploitation of microalgal bioactive compounds.

## Figures and Tables

**Table 1:** Average extract content (mean ± standard deviation) in fatty acids (μg_FattyAcids_·mg_Biomass_^−1^) (*n* = 6), carotenoids expressed in equivalent of PI (trans-β-Apo-8′-carotenal), mg_PI_·mg_Biomass_^−1^ (*n* = 6) and antioxidant capacity expressed in the trolox equivalent (TE), mg_TE_·mg_Extract_^−1^. mg_Biomass_^−1^ (*n* = 9), obtained at each amount of biomass tested (50, 100 and 150 mg), at 40 °C and under an ethanol flow-rate of 2 mL·min^−1^ (P = 142 bar). Better results pertaining to extraction of each particular compound are marked in bold.

		Biomass (mg)		
		50	100	150
**Fatty acids** (μg_FA_·mg_B_^−1^)	**14:0**	5.4 ± 0.6 ^a^	4.9 ± 1.0 ^a^	4.4 ± 0.5 ^a^
	**16:0**	24.6 ± 3.9 ^a^	23.0 ± 0.7 ^a^	23.3 ± 2.2 ^a^
	**18:0**	0.3 ± 0.0 ^a^	0.5 ± 0.1 ^a^	0.1 ± 0.0 ^a^
	**18:1 n9**	17.6 ± 3.0 ^a^	12.3 ± 3.7 ^a^	13.3 ± 2.5 ^a^
	**18:2 n6 t**	19.8 ± 3.6 ^a^	14.6 ± 1.0 ^a^	18.8 ± 2.0 ^a^
	**18:2 n6 c**	0.3 ± 0.0 ^a^	0.0 ± 0.0 ^a^	0.3 ± 0.0 ^a^
	**18:3 n6**	**0.9 ± 0.1**	0.5 ± 0.0 ^a^	0.4 ± 0.0 ^a^
	**18:3 n3**	36.6 ± 5.4 ^a,b^	27.1 ± 2.7 ^b^	39.3 ± 3.3 ^a^
**Carotenoids** (mg_PI_·mg_B_^−1^)	**Lutein**	**2.16 ± 0.14**	1.15 ± 0.06	1.39 ± 0.18
	**β-carotene**	0.16 ± 0.00 ^a^	0.13 ± 0.02 ^a^	0.12 ± 0.01 ^a^
**Antioxidant capacity** (mg_TE_·mg_E_^−1^. mg_B_^−1^)	**ABTS^+•^**	**129.4 ± 2.6**	102.7 ± 8.0	70.1 ± 5.5
	**DPPH^•^**	**3.1 ± 0.1**	1.9 ± 0.4 ^a^	1.9 ± 0.5 ^a^

^a–b^ Means within the same row, without a common superscript, are significantly different (p < 0.05).

**Table 2:** Comparison of results (mean ± standard deviation) obtained during optimization of Continuous pressurized ethanol extraction system (CPSE), with ultrasound-assisted extraction (UAE). Better results pertaining to extraction of each particular compound are marked in bold.

		Extraction Methods Tested	
		UAE	Optimized Conditions of CPSE
**Fatty acids** (μg_FA_·mg_E_^−1^)	**14:0**	9.9 ± 1.4 ^a^	10.0 ± 1.9 (5C) ^a^
	**16:0**	38.5 ± 4.1	50.8 ± 2.5 (5C)
	**16:1**	2.9 ± 0.2 ^a^	2.9 ± 1.5 (5C) ^a^
	**18:0**	0.9 ± 0.2 ^a^	1.5 ± 0.6 (5C) ^a^
	**18:1 n9**	23.5 ± 0.9	**33.8 ± 8.7** (5C) *
	**18:2 n6 t**	18.8 ± 2.0 ^a^	23.8 ± 5.2 (5C) ^a^
	**18:2 n6 c**	4.8 ± 0.1 ^a^	1.8 ± 1.1 (5C) ^a^
	**18:3 n6**	6.1 ± 1.1	3.3 ± 1.3(5C)
	**18:3 n3**	2.9 ± 0.1	**27.9 ± 4.2** (5C)
**Carotenoids** (mg_PI_·mg_E_^−1^)	**Lutein**	1.22 ± 0.18	**2.9 ± 0.1** (3C)
	**β-Carotene**	0.10 ± 0.01 ^a^	**1.5 ± 0.1** (3C)
**Antioxidant capacity** (µg_TE_·mg_E_^−1^)	**ABTS^+•^**	121.6 ± 6.2	**168.7 ± 4.3** (3C)
	**DPPH^•^**	395.1 ± 10.9	**423.4 ± 10.6** (3C)

^a–c^ Means within the same row, without a common superscript, are significantly different (*p* < 0.05; * *p* < 0.01).

**Table 3:** Conditions and operating conditions tested at all stages of CPSE optimization.

Stage		Abbreviation	Condition Tested	Operating Conditions
1	Biomass	M50	50 mg DW_biomass_	Q: 2 mL·min^−1^ T: 40 °C V_ethanol_: 150 mL
		M100	100 mg DW_biomass_	
		M150	150 mg DW_biomass_	
2	Flow	Q1	1 mL·min^−1^	DW_biomass_: 50 mg T: 60 °C V_ethanol_: 150 mL
		Q2	2 mL·min^−1^	
		Q3	3 mL·min^−1^	
		Q4	4 mL·min^−1^	
3	Temperature	T30	30 °C	DW_biomass_: 50 mg Q: 3 mL·min^−1^ V_ethanol_: 150 mL
		T40	40 °C	
		T50	50 °C	
		T60	60 °C	
		T70	70 °C	
4	Fractions of total solvent volume	1F	1st fraction of 12.5 mL	DW_biomass_: 50 mg Q: 3 mL·min^−1^ T: 60 °C
		2F	2nd fraction of 12.5 mL	
		3F	3rd fraction of 125 mL	
	Cycles of solvent recirculation	1C (1F)	1 cycle; 4 min	DW_biomass_: 50 mg Q: 3 mL·min^−1^ T: 60 °C V_ethanol_: 12.5 mL
		2C	2 cycles; 8 min	
		3C	3 cycles; 12 min	
		4C	4 cycles; 16 min	
		5C	5 cycles; 20 min	

## Supplementaly Materials

The following are available online at http://www.mdpi.com/1660-3397/16/9/327/s1.
