# Supplementary material for: Gloeothece sp. as a Nutraceutical Source—An Improved Method of Extraction of Carotenoids and Fatty Acids
Source: Mar Drugs. 2018 Sep 11;16(9):327. doi: 10.3390/md16090327 (PMC6163995; doi:10.3390/md16090327)
Supplement: Supplementary file 1 [file marinedrugs-16-00327-s001.pdf]

marine drugs

Supplementary Data

Gloeothece sp. as a Nutraceutical Source—An Improved Method of Extraction of Carotenoids and Fatty Acids

Helena M. Amaro [1,2], A. Catarina Guedes [1,*], Marco A. C. Preto [1], I. Sousa-Pinto [1,3] and F. Xavier Malcata [4,5]

[1] Interdisciplinary Centre of Marine and Environmental Research (CIIMAR), University of Porto, Terminal de Cruzeiros do Porto de Leixões, Avenida General Norton de Matos, S/N, P-4450-208 Matosinhos, Portugal; lena.amaro@gmail.com (H.M.A.); mcpreto@gmail.com (M.A.C.P.); ispinto@ciimar.up.pt (I.S.-P.)
[2] Institute of Biomedical Sciences Abel Salazar (ICBAS), University of Porto, Rua Jorge Viterbo Ferreira no. 228, P-4050-313 Porto, Portugal
[3] FCUP—Faculty of Sciences, University of Porto, Rua do Campo Alegre s/n, 4169-007 Porto, Portugal
[4] LEPABE—Laboratory of Process Engineering, Environment, Biotechnology and Energy, Rua Dr. Roberto Frias s/n, P-4200-465 Porto, Portugal
[5] Department of Chemical Engineering, University of Porto, Rua Dr. Roberto Frias, s/n 4200-465 Porto, Portugal; fmalcata@fe.up.pt
* Correspondence: acatarinaguedes@gmail.com; Tel.: +351-22-340-18-06; Fax: +351-22-339-06-08

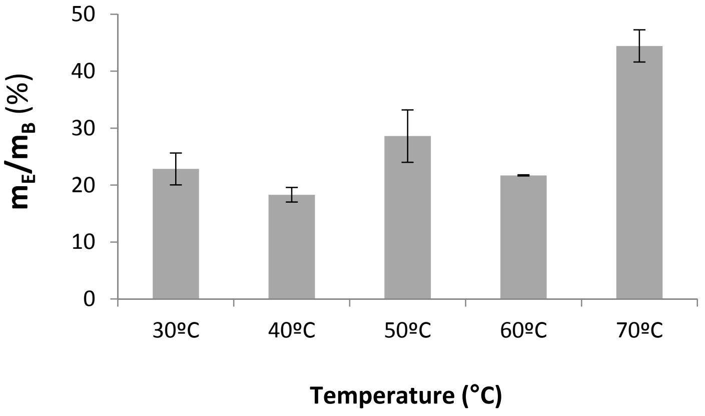

Figure S1. Extracts percentage of mass extract obtained at the different temperatures tested, obtained from 50 mg biomass, at solvent flow of Q3.

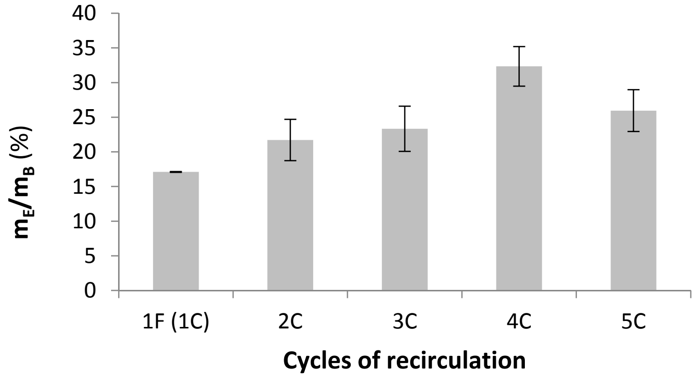

Figure S2. Extracts percentage of mass extract (m_E/m_B (%)) obtained at the different cycles or solvent recirculation tested, using from 50 mg biomass with solvent flow of Q3 ate 60ºC.
